# Modifying Surface Charges of a Thermophilic Laccase Toward Improving Activity and Stability in Ionic Liquid

**DOI:** 10.3389/fbioe.2022.880795

**Published:** 2022-06-08

**Authors:** Joseph C. Stevens, Jian Shi

**Affiliations:** Biosystems and Agricultural Engineering, University of Kentucky, Lexington, KY, United States

**Keywords:** lignin, ionic liquid, lignin degrading enzymes, surface modifacation, docking simulation

## Abstract

The multicopper oxidase enzyme laccase holds great potential to be used for biological lignin valorization alongside a biocompatible ionic liquid (IL). However, the IL concentrations required for biomass pretreatment severely inhibit laccase activity. Due to their ability to function in extreme conditions, many thermophilic enzymes have found use in industrial applications. The thermophilic fungal laccase from *Myceliophthora thermophila* was found to retain high levels of activity in the IL [C_2_C_1_Im][EtSO_4_], making it a desirable biocatalyst to be used for lignin valorization. In contrast to [C_2_C_1_Im][EtSO_4_], the biocompatibility of [C_2_C_1_Im][OAC] with the laccase was markedly lower. Severe inhibition of laccase activity was observed in 15% [C_2_C_1_Im][OAc]. In this study, the enzyme surface charges were modified *via* acetylation, succinylation, cationization, or neutralization. However, these modifications did not show significant improvement in laccase activity or stability in [C_2_C_1_Im][OAc]. Docking simulations show that the IL docks close to the T1 catalytic copper, likely interfering with substrate binding. Although additional docking locations for [OAc]^-^ are observed after making enzyme modifications, it does not appear that these locations play a role in the inhibition of enzyme activity. The results of this study could guide future enzyme engineering efforts by showing that the inhibition mechanism of [C_2_C_1_Im][OAc] toward *M. thermophila* laccase is likely not dependent upon the IL interacting with the enzyme surface.

## Introduction

Lignin is among the most abundant terrestrial biopolymers that make up ∼25% of the weight of plant biomass (Weng and Chapple, 2010; Doherty et al., 2011). It is primarily made of three phenylpropanoid subunits: *p*-hydroxyphenyl (H), guaiacyl (G), and syringyl (S) that can be linked together *via* several condensed (C-C) or ether (C-O) bonds, with the phenylcoumaran (β – 5), β-aryl ether (β – O – 4), and pinoresinol (β – β) being the most common (Boerjan et al., 2003; Vanholme et al., 2010; Sette et al., 2011; Zeng et al., 2013). In addition, the ratio of subunits present in the lignin network is dependent upon the source of the biomass (Suhas et al., 2007). As a result of lignin’s structural and monomeric heterogeneity, thermochemical and biological methods achieve poor selectivity and yield of breakdown products. Lignin is currently generated in high volumes as a waste product by the paper and pulping industry and during production of cellulosic biofuels (National Academy of Sciences, National Academy of Engineering, and National Research Council, 2009; Tilman et al., 2009). Converting lignin from a waste product to value added chemicals *via* new technologies will not only reduce waste lignin accumulation but also add value to the paper and pulping and future cellulosic biorefineries (Ragauskas et al., 2014; Mottiar et al., 2016). While the applications of polymeric lignin are limited, the phenolic products obtained from lignin deconstruction can be used as fuels or precursors for chemicals in the food, pharmaceutical, or plastic industries; many of these fuels and chemicals are currently derived from petroleum (Kleinert and Barth, 2008; Cotoruelo et al., 2011).

Current methods for lignin deconstruction can be divided into thermochemical (e.g., high temperature pyrolysis, catalytic oxidation, and hydrogenolysis) and biological (e.g., lignin degrading enzymes [LDEs] or microbes) (Laskar et al., 2013; De Wild et al., 2014; Beckham et al., 2016; He et al., 2017). Much of the research into the thermochemical methods has focused on improving the catalyst performance at high temperatures and improving the selectivity of lignin-derived products (Das et al., 2017; Li et al., 2017; Das et al., 2018). The use of LDEs in biological lignin deconstruction strategies could enable lignin deconstruction at lower temperatures and improve product selectivity due to the inherent selectivity and efficiency of biocatalysts (Beckham et al., 2016). Many LDEs such as the peroxidases (lignin, manganese, and versatile) and laccases have been discovered in bacteria, plant, and fungi (Hullo et al., 2001; Miyazaki, 2005; An et al., 2015). In addition, catabolic lignin pathways in soil bacteria, such as *Sphingobium* sp. SYK-6 that catabolizes lignin using NAD or glutathione-dependent enzymes have recently been identified (Varman et al., 2016). The application of LDEs for lignin deconstruction has been limited by a number of factors, chief among these being the high cost of enzyme production and the low yield of products due to the poor solubility of lignin in solvents biocompatible with LDE activity (Bugg et al., 2011; Brown and Chang, 2014).

Many of the known lignin solvents (e.g., dimethyl sulfoxide [DMSO] and alkaline solutions) reduce or altogether eliminate enzyme activity (Mozhaev et al., 1989; Klibanov, 2001). In addition, these solvents require high temperatures to facilitate the complete dissolution of plant derived lignin, leading to a further loss in enzyme activity (Park and Kazlauskas, 2003). Therefore, a solvent system that is capable of solubilizing lignin at low temperatures and mild pH is desirable. Ionic liquids (ILs) are molten, organic salts liquid at temperatures <100°C, the properties of which can be tuned by selecting the appropriate cation and anion (Hajime et al., 2000; Brennecke and Maginn, 2001; Gutowski et al., 2003; Rogers and Seddon, 2003). The alkylimidazolium ILs, such as 1-ethyl-3-methylimidazolium acetate ([C_2_C_1_Im][OAc]) and 1-butyl-3-methylimidazolium chloride ([C_4_C_1_Im][Cl]), have been the focus of numerous studies on biomass pretreatment at relatively low temperatures (Fort et al., 2007; Kilpeläinen et al., 2007; Verdía et al., 2014). Due to the high cost associated with using these ILs, recent efforts have focused on developing low-cost ILs from bio-derived cations (e.g., choline and ammonium) and anions (e.g., carboxylic acids and amino acids) that can be used in low concentrations during pretreatment (Socha et al., 2014; George et al., 2015; Gschwend et al., 2018). One example is cholinium lysinate ([Ch][Lys]) which, at only 10% (w/v) in water, was shown to remove 80% of lignin from genetically engineered switchgrass during pretreatment (Liu et al., 2017).

Laccases are a member of the multicopper oxidase superfamily of enzymes (E.C. 1.10.3.2). They were first discovered in extract from the Japanese lacquer tree (*Toxicodendron vernicifluum*) in 1883 (Yoshida, 1883). Laccases differ from the other group of lignolytic enzymes, the heme peroxidases, in that they are copper-containing enzymes that do not require the presence of a strong oxidant, such as hydrogen peroxide, for the reaction mechanism (Glenn et al., 1983; Piontek et al., 2002). The substrate is oxidized at the T1 catalytic copper *via* 4-electron removal, after which the electrons are shuttled 13Å to the trinuclear copper cluster where molecular oxygen is reduced to water (Jones and Solomon, 2015). The T1 catalytic copper and the trinuclear coppers (a T2 and 2 T3) are coordinated with four conserved HXH motifs; X = cysteine in the case of the T1 copper (Piontek et al., 2002). The T1 copper is also coordinated by methionine or leucine/phenylalanine in bacterial/plant and fungal laccases, respectively (Claus, 2004).

Laccases are also capable of oxidizing nonphenolic lignin compounds when used alongside a small molecule mediator, such as 1-hydroxybenzotriazole (HBT) or 2,2′-azino-bis(3-ethylbenzthiazoline-6-sulfonic acid) (ABTS) (Gamelas et al., 2005; d’Acunzo et al., 2006; Rico et al., 2014). The relatively mild reaction conditions required for laccase activity along with the oxidation of mediator compounds make laccases a desirable biocatalyst for use in many biotechnological applications, including biological lignin deconstruction strategies (Singh Arora and Kumar Sharma, 2010; Mate and Alcalde, 2015). Many ILs have been shown to be biocompatible with the activity of laccases at low concentrations in water. When Galai *et al.* investigated the effect of 56 ILs on the activity of *T. versicolor* laccase for dye decoloring applications, they found that 13 of the ILs increased the activity of the laccase; 10 mM choline dihydrogen phosphate ([Ch][H_2_PO_4_]) increased activity by 451% (Galai et al., 2015). The initial activity of laccase from the white rot fungus, *Trametes versicolor,* was only reduced by 20% in a reaction mixture containing 50% (v/v) 1-ethyl-3-methylimidazolium ethylsulfate ([C_2_C_1_Im][EtSO4]) (Domínguez et al., 2011). However, other alkylimidazolium ILs have been found to be less biocompatible with laccase activity. The initial activity of *T. versicolor* laccase was reduced by 50% in reaction mixtures containing just 3, 20, 40, and 5% (v/v) of [C_2_C_1_Im][OAc], [C_4_C_1_Im][Cl], 1-hexyl-3-methylimidazolium bromide ([C_6_C_1_Im][Br]), and 1-decyl-3-methylimidazolium chloride ([C_10_C_1_Im][Cl]), respectively (Domínguez et al., 2011; Stevens et al., 2019).

Recent studies have shown that ILs are more biocompatible with thermophilic enzymes compared to mesophilic enzymes. Hydrolysis of carboxymethylcellulose (CMC) by hyperthermophilic (T_opt_ > 95°C) and thermophilic (T_opt_ = 80°C) cellulases was minimally impacted in 0–20% (v/v) [C_2_C_1_Im][OAc] while a mesophilic cellulase (T_opt_ = 37°C) was unable to hydrolyze CMC in only 10% (v/v) [C_2_C_1_Im][OAc] (Datta et al., 2010). Cellulases and xylanase secreted by *Galactomyces sp.* showed improved tolerance to IL [Mmim][DMP] and effective hydrolysis of chestnut shell (He et al., 2016). Previous work also demonstrated integration of biocatalyst, for example, ω-transaminase and l-alanine dehydrogenase into a chemoenzymatic process to convert biomass to valuable products such as furfurylamine (Li et al., 2021; Ni et al., 2021). The activity of laccase from the thermophilic fungi *Myceliophthora thermophila* (*MtL*) is increased 3-fold in 25% (v/v) [C_2_C_1_Im][EtSO_4_] (Fernández-Fernández et al., 2014). The bacteria *Bacillus subtilis* produces a laccase-like spore coat protein (CotA) that can oxidize canonical laccase substrates ABTS and syringaldazine (SGZ) in the presence of several alkylimidazolium chloride ([C_n_C_1_Im][Cl]) ILs [C_n_C_1_Im][Cl] ILs have been shown to severely inhibit the activity of a mesophilic laccase at low concentrations (Hullo et al., 2001; Dabirmanesh et al., 2015). However, we showed in a previous study that the most thermophilic laccase identified to date, produced by the hyperthermophilic bacterium *Thermus thermophilus*, is highly sensitive to low concentrations (<10% v/v) of [C_2_C_1_Im][OAc], suggesting that enzyme thermophilicity alone does not guarantee high activity in ILs (Miyazaki, 2005).

In this study we sought to determine the biocompatibility of aqueous [C_2_C_1_Im][OAc] with *MtL*. To do this, we first screened the activity of *MtL* in different concentrations of [C_2_C_1_Im][OAc] up to 50% in water. We also measured the effect of low concentrations of [C_2_C_1_Im][OAc] on the thermostability of *MtL*. Following the initial biocompatibility and stability screenings, we made several surface charge modifications with the aim of improving *MtL* activity and stability in [C_2_C_1_Im][OAc]. We also used molecular docking simulations to understand how [C_2_C_1_Im][OAc] docks to the surface of *MtL* and the four charge variants we produced. The results of this study expand our understanding of laccase-IL interactions and the difficulties faced when trying to improve enzyme activity in ILs.

## Materials and Methods

### Materials

Laccase from *M. thermophila* expressed in *Aspergillus oryzae* (Batch # OMN07029) was kindly provided by Novozymes (Bagsværd, Denmark). The 1-Step™ ABTS substrate solution was purchased from ThermoFisher Scientific (Waltham, MA). All other reagents, including the IL [C_2_C_1_Im][OAc], were purchased from MilliporeSigma (St. Louis, MO).

### Surface Charge Modifications

*MtL* was purified by sequentially spin filtering with 100 and 30 kDa MWCO filters at 8°C prior to surface charge modifications. Surface charges were modified according to previously reported protocols (Nordwald and Kaar, 2013; Nordwald et al., 2014), and the induced surface charge modifications were summarized in Table 1. Cationized *MtL* was produced by modification with 0.5 M ethylenediamine hydrochloride in 200 mM MES buffer (pH 4.5) containing a 20:1 M ratio of the crosslinking reagent *N*-(3-dimethylaminopropyl)-*N′*-ethylcarbodiimide EDC-to-acid sites for 2.5 h at room temperature; neutralized *MtL* was similarly produced using diethylamine in place of ethylenediamine hydrochloride. Succinylated *MtL* was produced by the addition of four aliquots of succinic anhydride to the enzyme in 1 M sodium carbonate buffer (pH 8.5) over 3 h at room temperature. Acetylated *MtL* was produced by the addition of three aliquots of acetic anhydride to the enzyme in 100 mM sodium phosphate buffer (pH 7.0) over 1.5 h at room temperature. The final molar ratio of acetic or succinic anhydride to enzyme containing primary amines was approximately 30:1. Excess reagent was removed with 30 kDa MWCO spin filters at 4°C and the enzymes were stored in a 100 mM sodium phosphate buffer (pH 7.0) prior to experiments. The protein content was measured using the Bradford assay.

**TABLE 1:** Summary of mutations to used mimic surface charge modifications in PyMol.

Charge variant	Modification chemistry	Target residues	PyMol mutations
Unmodified	N/A	N/A	N/A
Acetylated	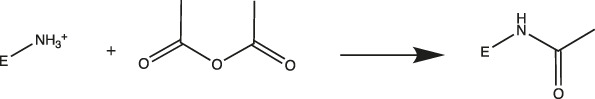	Primary Amines (Lys)>	Lys → Ala
Neutralized	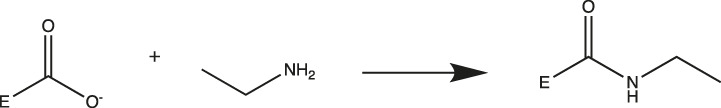	Carboxylic Acid (Asp or Glu)>	Asp → Ala
			Glu → Ala
Succinylated	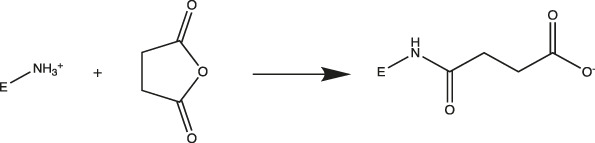	Primary Amines (Lys)>	Lys → Asp
Cationized	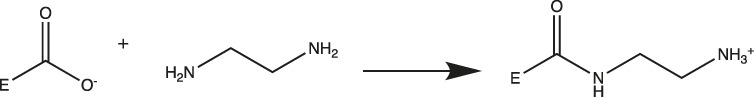	Carboxylic Acid (Asp or Glu)>	Asp → Lys
			Glu → Lys

### Biocompatibility Screening

The biocompatibility of [C_2_C_1_Im][OAc] in aqueous solution (approximately 1–50% w/v), was screened with all *MtL* variants. To reduce the effect of pH, the IL solution was adjusted to pH 4.5 using 1 M hydrochloric acid prior to testing. The activity was screened with a 50 mM citrate/100 mM phosphate buffer (pH 4.5), IL (0, 1, 2, 3, 4, or 10% w/v) and 50 μL ABTS solution in clear, flat bottom, 96-well Costar assay plates (Corning Inc., Kennebunk, ME). Absorbance readings were taken every 15 s for 10 min at 40°C in a SpectraMax M2 plate reader (Molecular Devices, Sunnyvale, CA). The plates were shaken for 3 s prior to each reading to ensure a well homogeneous solution. Oxidation of ABTS in the buffer and the AIL without laccase were measured as blanks. The activity of *MtL* in ILs relative to buffer was calculated using Eq. 1.

$$Relative\;activity=\;\frac{Initial\;Velocity\;in\;IL}{Initial\;Velocity\;in\;buffer}$$ (1)

### MtL Thermostability

To measure the thermostability of *MtL*, all charge variants were incubated at 40°C in 0, 2.5, or 5% [C_2_C_1_Im][OAc] and 50 mM citrate/100 mM phosphate buffer, pH 4.5. At each time interval (0, 30, 60, 120, 180, and 240 min) aliquots were removed and placed on ice prior to measuring residual enzyme activity. The activity was screened using the same method in *4.3.3. Biocompatibility Screening*. The residual activity of *MtL* and the charge variants after incubation was calculated using Eq. 2.

$$Residual\;activity=\;\frac{Initial\;Velocity\;after\;incubation}{Initial\;Velocity\;after\;0\min incubation}$$ (2)

### Docking Simulations

The 3D structures and PDB files of the ligands were prepared in YASARA Structure (YASARA Biosciences GmbH, Vienna, Austria) using the SMILES strings obtained from PubChem. The PDBQT files of the ligands and *MtL* (PDBID: 6F5K) were prepared with AutoDock (version 4.2.6, MGLTools, La Jolla, CA). Charges for the coppers were added by manually editing the *MtL* PDBQT file. AutoGrid parameters were as follows: space value of 0.375Å (x, y, z) grid centered at (4.840, 28.290, -18.567), and grid size of 126 in all directions. AutoDock parameters were as follows: Lamarckian GA, 100 genetic algorithm runs, and 25,000,000 max eval size. Docking results were visualized using PyMol (Schrodinger LLC, New York, NY). To simulate the surface charge modifications, target surface residues were mutated in PyMol. These mutations are shown in Table 1.

## Results

### Biocompatibility Screening

**FIGURE 1:**
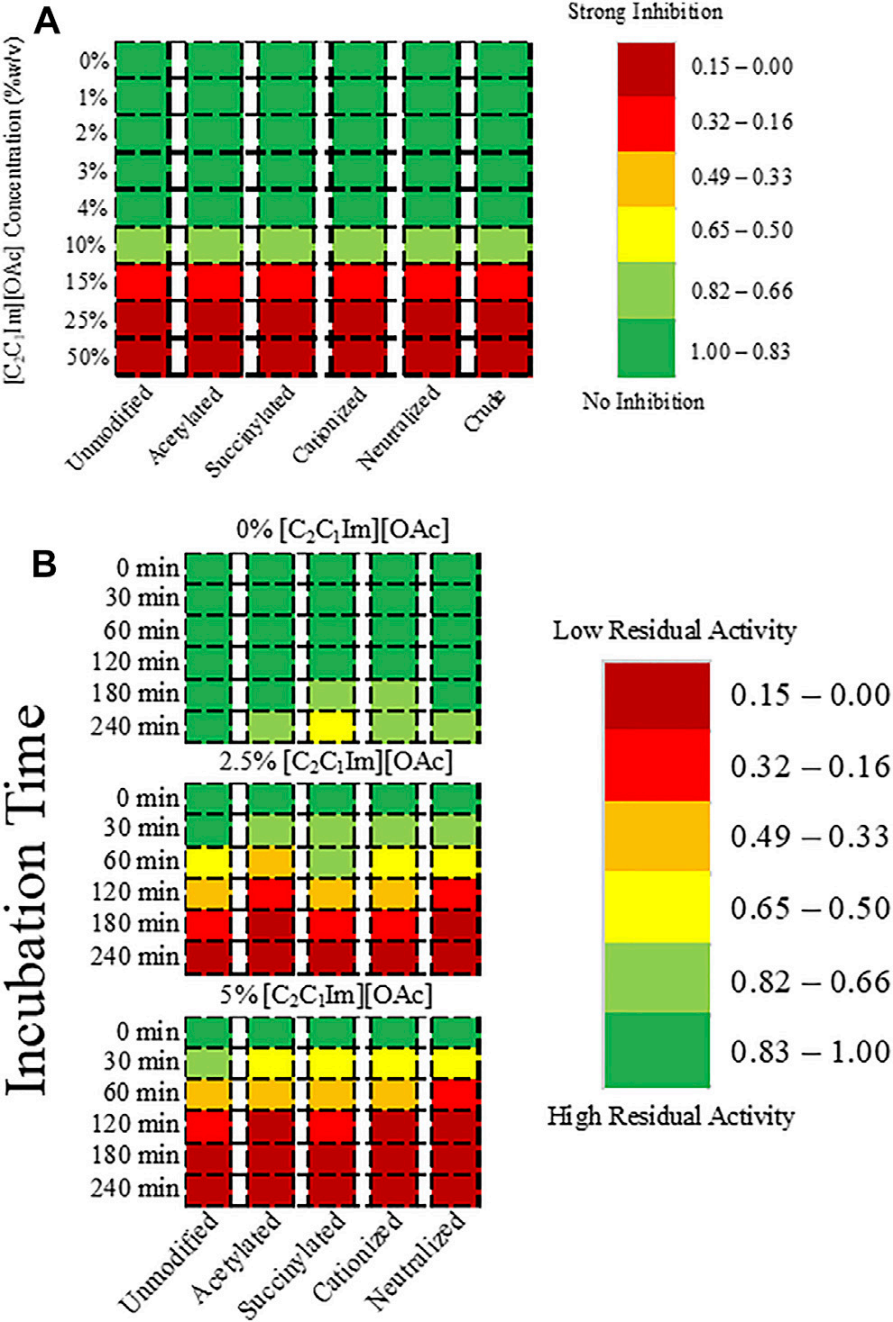
Heatmap showing **(A)** the activity of *MtL* and the charge variants in [C_2_C_1_Im][OAc] relative to *MtL* activity in buffer, and **(B)** the residual activity of *MtL* charge variants after incubation in 50 mM citrate/100 mM phosphate buffer and 2.5 or 5% [C_2_C_1_Im][OAc] at pH 4.5 and 40°C.

To understand the biocompatibility of *MtL* with [C_2_C_1_Im][OAc], we measured the activity of *MtL* and all of the charged variants in aqueous solutions (0–50% w/v) of [C_2_C_1_Im][OAc] in water. None of the surface charge modifications significantly improved the activity of *MtL* in aqueous [C_2_C_1_Im][OAc] relative to the unmodified *MtL* (Figure 1A). Minimal inhibition of *MtL* activity is observed in 1–10% [C_2_C_1_Im][OAc], however severe inhibition of *MtL* activity is observed in 15–50% [C_2_C_1_Im][OAc]. Since the coppers are not coordinated by any modifiable residues, it is unlikely that the inhibition is due to the loss of the coppers. The range of [C_2_C_1_Im][OAc] concentrations tested in this study is based on recent reports that reduced lignin removal, lower fermentable sugar yields, and lower cellulose solubilization are observed when less than 50% IL is present in aqueous solutions of [C_2_C_1_Im][OAc] and water (Shi et al., 2014).

The cation and anion of the IL play a role in determining the biocompatibility of the IL with enzyme activity. Previous studies have shown that as the alkyl chain length of the cation increases, alkylimidazolium ILs become more inhibitory (Domínguez et al., 2011; Dabirmanesh et al., 2015). The inhibitory effect of the anion has been shown to follow the Hofmeister series (Weingärtner et al., 2012; Patel et al., 2014). ILs with chaotropic anions (e.g., NTf_2_^-^ and Br^−^) more strongly destabilize proteins when compared to kosmotropic anions (e.g., SO_4_^2−^ and PO_4_^3−^). For example, the activity of *T. versicolor* laccase is increased by 451% in 10 mM choline dihydrogen phosphate ([Ch][H_2_PO_4_]), an IL with a kosmotropic anion, but is not affected by the same concentration of choline bis([trifluoromethyl]sulfonyl)imide ([Ch][NTf_2_]), an IL with a chaotropic anion (Galai et al., 2015). Nordwald et al. previously showed that acetylation and succinylation improve enzyme activity and stability in 1-butyl-3-methylimidazolium chloride ([C_4_C_1_Im][Cl]) by reducing the interaction of the chaotropic Cl^−^ anion with the enzyme surface. Additionally, low enzyme activity in higher concentrations of [C_2_C_1_Im][OAc] can be attributed to the buffering capacity of [C_2_C_1_Im][OAc]. Optimal ABTS oxidation occurs at pH < 5 for most fungal and bacterial laccases, but [C_2_C_1_Im][OAc] buffers at a pH well above this range thereby lowering *MtL* activity in high concentrations of [C_2_C_1_Im][OAc] (Vernekar and Lele, 2009).

### Stability in [C_2_C_1_Im][OAc]

Although the surface charge modifications did not improve the activity of *MtL*, we sought to understand if the modifications improved the stability of *MtL* in [C_2_C_1_Im][OAc]. All charge variants of *MtL* were incubated in 50 mM citrate/100 mM phosphate buffer (pH 4.5) with 0, 2.5, and 5% [C_2_C_1_Im][OAc] at 40°C and the activity was measured at different time intervals up to 4 h. The surface charge modifications, by following the established protocols successfully tested on enzymes like cellulase and lipase, did not improve the stability of *MtL* in low concentrations of [C_2_C_1_Im][OAc] (Figure 1B). Minimal activity loss was observed after 4 h in the buffer for all charge variants. When incubated in 2.5% [C_2_C_1_Im][OAc] >50% initial activity is lost after 2 h, while >50% initial activity is lost after only 1 h in 5% [C_2_C_1_Im][OAc]. The IL concentrations tested are based on the results of the biocompatibility screening: 2.5 and 5% [C_2_C_1_Im][OAc] represent ranges of IL concentrations in which no or minimal activity loss is observed, respectively.

Previously, enzymes with surface charge modifications in ILs were shown to have a higher or lower half-life relative to the wild type in the IL. We have summarized some of the previous studies using various methods to improve enzyme activity in ILs (Table 2). Among those, the surface modification method has been applied to lipase and cellulase, however, few reported surface modifications on laccases. Both succinylation and acetylation improved the half-life of lipase and α - chymotrypsin in 40% [C_4_C_1_Im][Cl] and 55% [C_2_C_1_Im][EtSO_4_], respectively, while acetylation improved the half-life of papain in 30% [C_4_C_1_Im][Cl]; however, neutralization and cationization reduced the half-life of lipase and α - chymotrypsin in IL, respectively (Nordwald and Kaar, 2013). Succinylated and acetylated enzymes are thought to be more stable in ILs due to two factors: their similarity to halophilic enzymes and the formation of salt bridges. Halophilic enzymes have a large number of acidic surface residues which attract water molecules, increasing protein hydration and preventing the enzyme from aggregating, while the salt bridges further increase enzyme stability in saline environments (Dym et al., 1995; Elcock and McCammon, 1998; Uthandi et al., 2010).

**TABLE 2:** Previous studies using various methods to improve enzyme activity in ILs.

Method	Enzyme	Outcome	Citation
Computationally assisted protein engineering	*T. versicolor* laccase	Triple and quadruple mutants with increased activity in [C_2_C_1_Im][EtSO_4_]	Wallraf et al. (2018)
Directed evolution	*T. versicolor* laccase	Double mutant with increased activity in [C_2_C_1_Im][EtSO_4_]	Liu et al. (2013)
Computationally assisted protein engineerin	*B. subtilis* laccase	Single mutants with increased catalytic efficiency (k_cat_/K_m_) in 3 [C_n_C_1_Im][Cl] ILs	Dabirmanesh et al. (2015)
Surface charge modification	Bovine pancreas α-chymotrypsin, *Carica papaya* papain, *Candida rugosa* lipase	Succinylation and acetylation improved activity and stability in [C_4_C_1_Im][Cl] and [C_2_C_1_Im][EtSO_4_]	Nordwald and Kaar (2013)
Surface charge modification	*Trichoderma reesei* cellulase	Succinylation improved cellulose hydrolysis in [C_4_C_1_Im][Cl]	Nordwald et al. (2014)
Immobilization	*M. thermophila* laccase	Immobilization on glyoxyl-agarose beads improved stability in [C_2_C_1_Im][EtSO_4_]	Fernández-Fernández et al. (2014)

### Docking Simulations

**FIGURE 2:**
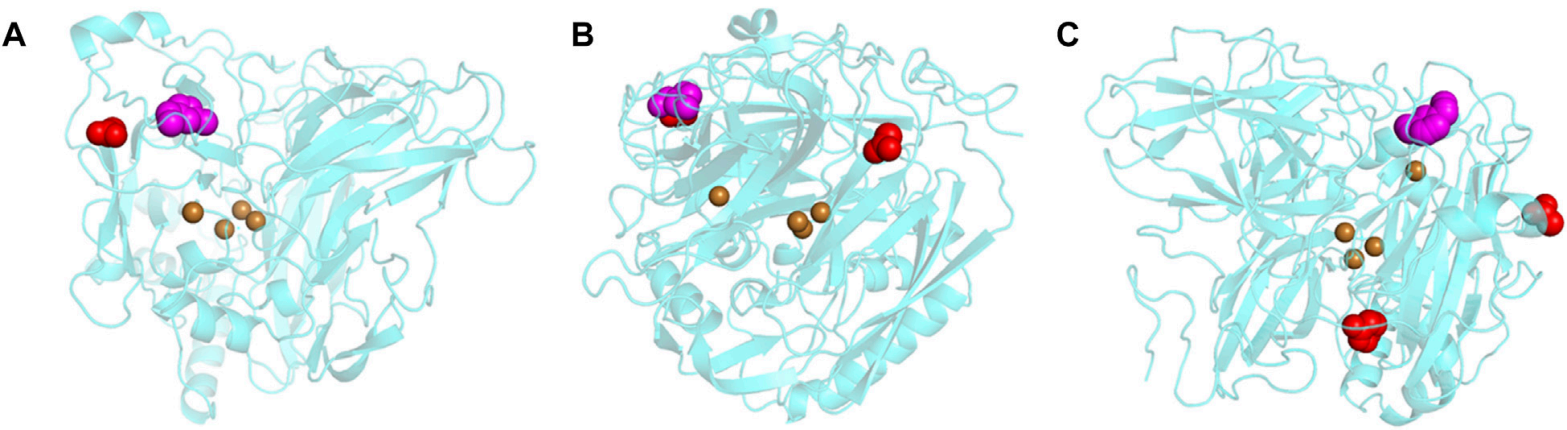
Major docking locations of [C_2_C_1_Im][OAc] to the surface of unmodified **(A)**, succinylated/acetylated **(B)**, and cationized/neutralized *MtL* **(C)** [C_2_C_1_Im]^+^ is shown as magenta spheres [OAc]^−^ is shown as red spheres, and coppers are shown as brown spheres.

Due to the unknown concentration of ABTS in the 1-Step solution used in the biocompatibility screening, we were unable to use enzyme kinetics analyses to understand the inhibition of *MtL* in [C_2_C_1_Im][OAc]. Therefore, docking simulations were used to better understand the effect of the surface charge modifications on the major binding locations of [C_2_C_1_Im][OAc] to the enzyme surface. The results of the docking simulations show that the modification chemistry affects the major docking locations of the IL to the surface of *MtL* (Figure 2). The major docking location of the IL to the surface of the unmodified *MtL* was limited to a region close to the T1 copper (Figure 2A). When *MtL* was acetylated or succinylated the IL docked close to the T1 copper in addition to a new [OAc]^-^ binding location away from the T1 copper (Figure 2B). When *MtL* was cationized or neutralized [C_2_C_1_Im]^+^ docked close to the T1 copper while [OAc]^-^ docked further from the T1 copper but still close to the active site entrance (cationized) or away from the active site (cationized and neutralized) (Figure 2C). In order to check whether the grid box size affects the docking simulation results, we have run additional simulations focusing the grid on the T1 copper + active site, a much smaller volume compared to our original simulation (Supplementary Figure S1). We found all of the main binding locations were close to the T1 copper or in the active site. This confirms the results obtained on a grid box covering the entire protein.

A closer inspection of the [C_2_C_1_Im]^+^ docking location reveals the presence of several aromatic residues that would not be affected by any of the surface charge modifications. These aromatic residues would be able to stabilize [C_2_C_1_Im]^+^ *via* π–π or π–cation interactions in all charge variants. While these results are consistent with a previous report that [C_2_C_1_Im]^+^ docks to *MtL* in the presence of aromatic residues, we did not observe [C_2_C_1_Im]^+^ entering the active site (Sun et al., 2017). This study did not restrict the possible [C_2_C_1_Im]^+^ binding locations, whereas the previous study focused on the docking around the *MtL* active site which could affect the degree to which the cation entered into the active site. Arg456 and His457 stabilize [OAc]^-^ close to the active site of unmodified, succinylated, and acetylated *MtL*. When docking to succinylated and acetylated *MtL* [OAc]^-^ is stabilized by Arg226 and Arg228 at the second [OAc]^-^ binding site. The major [OAc]^-^ docking location close to the active site of cationized *MtL* contains two modified residues (Asp360 and Glu420), in addition to several lysines. The second [OAc]- binding site to cationized and neutralized *MtL* contains several arginines, lysines, and modified residues.

The shift in [OAc]^-^ binding is determined by the modification chemistry. Acetylation and succinylation reduce the positive surface charges that stabilize [OAc]^-^, driving the anion to the arginine-rich binding pocket. Neutralization and cationization further increase the favorability of anion binding to an arginine-rich pocket containing modified glutamate and aspartate. The previous work on modifying the surface charges suggest that acetylation and succinylation discourage the chaotropic anion (e.g., Cl^−^ or [OAc]^-^) from interacting with the enzyme surface, thereby improving enzyme activity and stability in IL (Nordwald and Kaar, 2013; Nordwald et al., 2014). Our docking results show that all surface charge modifications encourage [OAc]^-^ binding away from the active site, however neither *MtL* activity nor stability in [C_2_C_1_Im][OAc] is affected by the modifications. Laccase activity is dependent upon the presence of a T1 copper in the active site, a trinuclear copper cluster, and a Cys-His pathway between the T1 copper and the trinuclear cluster. It is speculated that the loss of *MtL* activity and stability in [C_2_C_1_Im][OAc] is likely due to the destabilization of the coppers and the Cys-His pathway by the IL *via* a mechanism that cannot be influenced by modifying the enzyme surface charges. Further investigation of the surface properties of modified enzymes and enzyme kinetics will help to validate the proposed mechanism.

## Discussion

In this study, we examined the biocompatibility of dilute [C_2_C_1_Im][OAc] with the activity of the thermophilic fungal laccase from *Myceliophthora thermophila*. Results show severe inhibition of *MtL* activity in only 15% (w/v) [C_2_C_1_Im][OAc], far below the IL concentration necessary for effective biomass pretreatment. In an effort to reduce this inhibition of *MtL* activity, we made several surface charge modifications to increase the acid or amine surface residues. Although previous studies have shown that surface charge modifications can increase enzyme activity and stability in aqueous ILs, the stability and activity of all *MtL* charge variants in [C_2_C_1_Im][OAc] were unchanged compared to the unmodified enzyme. Docking simulations show that the IL docks close to the T1 copper on all charge variants, with some additional [OAc]^-^ docking locations on the surface of modified enzymes. Further work might seek to 1) optimize the surface charge modification method on laccase and investigate the surface properties of the modified enzyme, 2) better understand the mechanism by which the IL inhibits *MtL* activity through the use of enzyme kinetics and molecular dynamics (MD) simulations and 3) use additional techniques (e.g., rational design or immobilization) to improve *MtL* activity in [C_2_C_1_Im][OAc].

Methods used to assess the effects of ILs on enzymes can be divided into *in vitro* (e.g., activity assays, stability assays, and enzyme kinetics) and *in silico* [e.g., docking simulations and molecular dynamics (MD) simulations] techniques. *In vitro* techniques can be used to provide a broad overview of how the ILs are affecting the enzyme activity, stability, and substrate affinity. By measuring the activity of *TvL* in ILs, past studies have shown that [C_2_C_1_Im][EtSO_4_] is the most biocompatible alkylimidazolium IL, suggesting that this laccase-IL combination would be best suited for lignin deconstruction applications (Domínguez et al., 2011; Fernández-Fernández et al., 2014). However, these activity assays are done on a short time scale which does not provide any information about the stability of the laccase in the IL. Although the initial activity of *MtL* immobilized on glyoxyl-agarose beads is lower in 25% [C_2_C_1_Im][EtSO_4_] than the free laccase initial activity, the immobilized laccase is far more stable in 75% IL than the free laccase over a 7 days period (Fernández-Fernández et al., 2014). Enzyme kinetic studies, performed by measuring the initial velocity of enzymes in different substrate concentrations, can further indicate if the loss in activity is due to the IL affecting the enzyme activity or substrate binding. Kinetic studies of *MtL* in [C_2_C_1_Im][Cl] showed that the IL is a competitive inhibitor that competes with the substrate for binding in the active site (Sun et al., 2017) [C_2_C_1_Im][EtSO_4_] was found to interact with *MtL* as an uncompetitive inhibitor that does not affect substrate binding, but rather affects substrate oxidation once it has bound (Fernández-Fernández et al., 2014). Some ILs are also able to affect both substrate binding and oxidation, for example [C_2_C_1_Im][OAc] and [Ch][Lys], which were found to be mixed inhibitors that decreased substrate affinity and activity of *TvL* (Stevens et al., 2019). In contrast, several studies had demonstrated synergy between IL and biocatalyst and the promotion of enzyme activity in IL. For example, Galai et al. found that 13 ILs increased the activity of the *T. versicolor* laccase with the most significant improvement (451%) seen in 10 mM choline dihydrogen phosphate ([Ch][H_2_PO_4_]) (Galai et al., 2015). The authors attribute a shift to the α-helix structure induced by [Ch][H_2_PO_4_] that could be responsible for the enhancement of the enzyme activity. A synergetic effect was also observed between 1-butyl-2,3-dimethylimidazolium cetyl-PEG sulfate and lipase catalyzed transesterification of secondary alcohols (Yoshiyama et al., 2013).

To further understand the laccase-IL interactions seen with kinetic studies, recent studies have used docking simulations with ILs and static protein models. Docking simulations showed that the diffusion of [C_n_C_1_Im]^+^ cations (*n* = 2, 4, 6, 8, 10) into the active site of *MtL* is determined by the alkyl chain length (Sun et al., 2017). Similarly, [C_2_C_1_Im][OAc] and [Ch][Lys] were found to dock close to the T1 copper of *TvL* (Stevens et al., 2019). In both of these cases the docking simulations supported the previous kinetic results showing the ILs can affect substrate binding and oxidation. MD simulations can also be used to better capture the dynamic nature of enzyme-IL interactions. After *in vitro* experiments showed [C_2_C_1_Im][OAc] to be more biocompatible with thermophilic cellulases than mesophilic cellulases, MD simulations revealed the IL effect is limited to local disturbances of thermophilic cellulases or secondary structure unfolding of mesophilic cellulases (Datta et al., 2010; Jaeger et al., 2015). MD simulations also provided further insight into enzyme-IL interactions that increase the thermostability of a *Fusarium solani* serine protease cutinase in 1-butyl-3-methylimidazolium hexafluorophosphate ([C_4_C_1_Im][PF_6_]) (Micaêlo and Soares, 2008). Further insight into the effect of the IL on enzymes can be gained by applying different methods to understand the binding-free energy of the IL or substrate to the enzyme surface or active site (Limongelli, 2020).

In this study we applied a single enzyme engineering approach to try and improve *MtL* activity and stability in [C_2_C_1_Im][OAc]. The enzyme used in this study was not produced recombinantly, therefore we were unable to apply biotechnological (e.g., directed evolution or rational design) methods to improve *MtL* activity and stability. Future studies with *MtL* in [C_2_C_1_Im][OAc] might apply immobilization techniques similar to those previously used to improve stability in [C_2_C_1_Im][EtSO4] (Fernández-Fernández et al., 2014). , recombinant expression of *MtL* would enable future researchers to apply other enzyme engineering techniques. The L1 loop can be targeted for mutations with a directed evolution or computationally assisted strategy, similar to previous studies with *TvL* (Liu et al., 2013; Wallraf et al., 2018). In addition, novel targets for mutations can be identified using a computationally assisted approach like that used to identify the Glu170 target in *B. subtilis* CotA (Dabirmanesh et al., 2015). The application of more robust methods for analyzing laccase-IL interactions alongside targeted enzyme engineering strategies will facilitate the identification of laccase-IL combinations suited for use in future lignin valorization systems.

## Data Availability

The raw data supporting the conclusion of this article will be made available by the authors, without undue reservation.
