# Supplementary material for: Modifying Surface Charges of a Thermophilic Laccase Toward Improving Activity and Stability in Ionic Liquid
Source: Front Bioeng Biotechnol. 2022 Jun 8;10:880795. doi: 10.3389/fbioe.2022.880795 (PMC9213733; doi:10.3389/fbioe.2022.880795)
Supplement: Supplementary file 1 [file DataSheet1.docx]

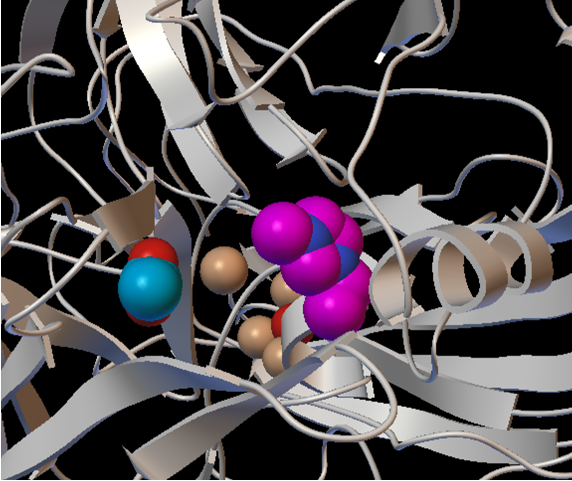

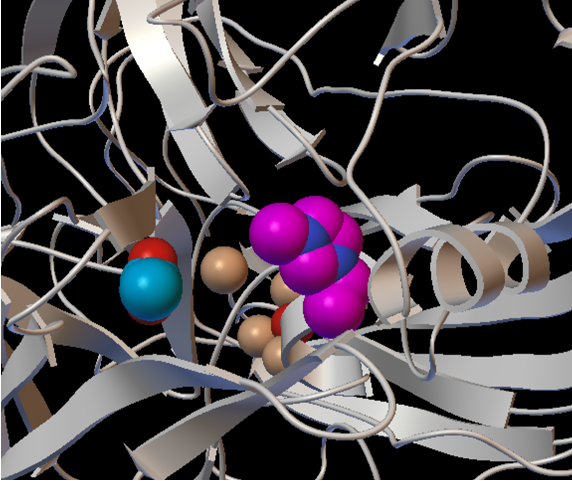


**b)**

**a)**


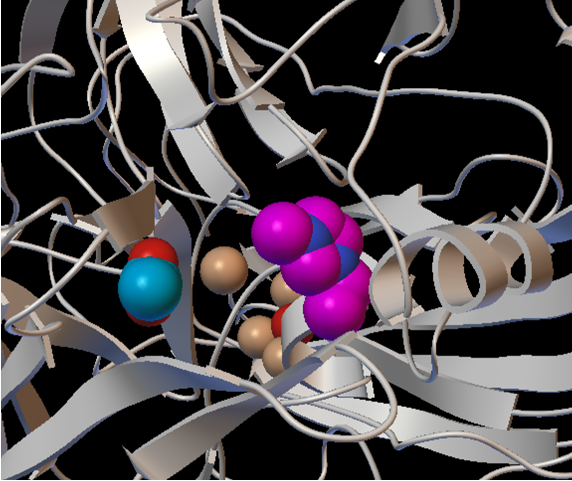

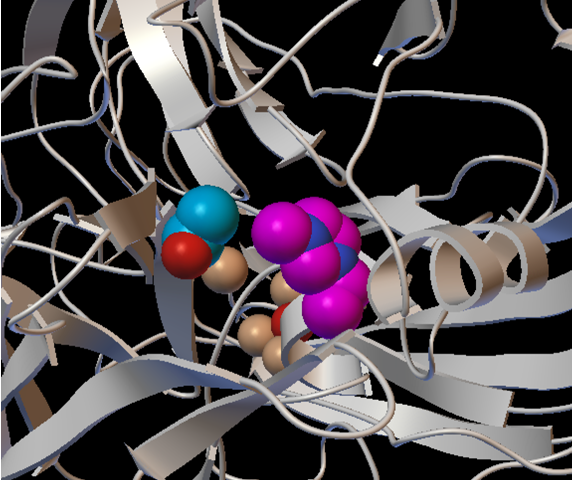


**d)**

**c)**


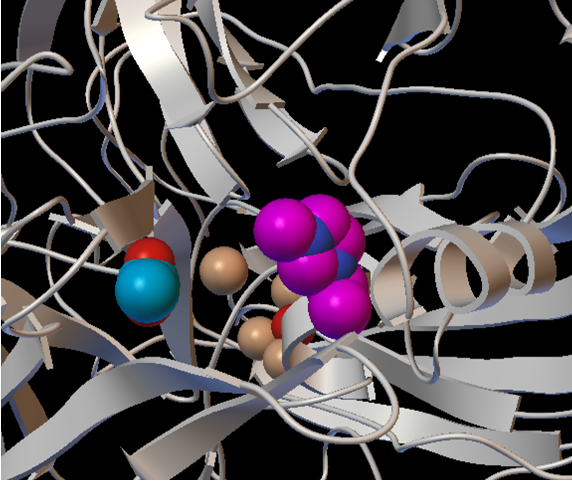


**e)**

**Figure S1**. Docking simulations on a smaller grid box covering T1 copper + active site. Major docking locations of [C_2_C_1_Im][OAc] to the surface of **a)** unmodified, **b)** succinylated, **c)** neutralized, **d)** cationized, and **e)** acetylated *MtL*.
